# PReS13-SPK-1478: New developments in our care & understanding of JIA

**DOI:** 10.1186/1546-0096-11-S2-I1

**Published:** 2013-12-05

**Authors:** L Wedderburn

**Affiliations:** 1 UCL Institute of Child Health, London, UK

## 

Recent years have seen a rapid change in speed of translation from basic science into the clinic: the benefits of this are now being felt right across pediatric rheumatology.

There is an increasing awareness that our patients need first class science to be done to answer major questions and develop better treatments: in fact patients and their families often ask why we are not making faster progress, and wish to contribute to this work.

To this end more and more large collaborative studies and networks are growing in our specialty that enable all children to be part of such studies.

In this session we will review some of the most exciting novel developments that have happened in the year of 2012-2013 in basic science, including novel results from genetics, molecular and functional biology.

We will consider how these new findings relate to patients with chronic autoimmune and inflammatory diseases, and how those developments may translate to better care and outcomes for our patients.

## Disclosure of interest

None declared.

